# *Clostridioides difficile* Infection: A Room for Multifaceted Interventions

**DOI:** 10.3390/jcm9124114

**Published:** 2020-12-20

**Authors:** Nicola Petrosillo, Maria Adriana Cataldo

**Affiliations:** 1 Clinical and Research Department, National Institute for Infectious Diseases “Lazzaro Spallanzani”, Via Portuense, 292-00149 Rome, Italy; 2 Epidemiology and Preclinical Research Department, National Institute for Infectious Diseases “Lazzaro Spallanzani”, Via Portuense, 292-00149 Rome, Italy; adriana.cataldo@inmi.it

*Clostridioides difficile* (CD) continues to be the number one health care-associated infectious pathogen in the United States [1] and a major concern in Europe [2]. The current COVID-19 pandemic has somewhat obscured all the other existing infectious diseases, and in some cases has also reduced attention to those measures useful for preventing health care-associated infections (HAI) and infections by multidrug-resistant germs. There are, indeed, several reports on the inappropriate use of antimicrobials in COVID-19 patients, with a worrying crisis of antimicrobial stewardship programs, and on the break in infection prevention and control measures [3,4]. Both the conditions are drivers for the occurrence of CD infection (CDI) in the healthcare setting [5]. Additionally, in the last years, concern has also been raised on the increasing incidence of community-acquired (CA) cases [6].

Recent surveillance on CDI reported that approximately 20%–27% of all cases were CA [7,8,9]. A meta-analysis that examined globally reported rates of CDI incidence found an overall incidence rate of CA-CDI of 0.55/1000 admissions per year [10]. Of note, CA-CDI cohorts appear to be younger with lower comorbidity scores and less exposure to antibiotics [11].

The different epidemiological profile of patients with CA-CDI may cause under-ascertainment and under-reporting and consequently results in the detection of lower incidence rates of CA-CDI [10]. In fact, the “European, multi-centre, prospective bi-annual point prevalence study of *Clostridium difficile* Infection in hospitalised patients with Diarrhoea” (EUCLID) study that aimed to measure the underdiagnosis of CDI across Europe showed that the median age of the undiagnosed patients was significantly lower than that of the patients with CDI tested at their participating hospitals [12].

Regrettably, the CDI underdiagnosis is a recognized and relevant issue, either because of suboptimal laboratory diagnostic methods or because of absence of clinical suspicion, as reported in one quarter of CDI cases in the EUCLID study [12,13].

Regarding inadequate laboratory diagnosis, the same EUCLID study showed that only two-fifths of European hospitals reported using optimum methods for testing CDI. Five per cent of all samples tested had a false-positive result—i.e., were diagnosed with CDI at the participating hospital but the diagnosis was not confirmed by a test performed at the reference laboratory; 2% of samples had a false negative result [12].

The rate of underdiagnosis has a strong impact in many fields of CDI management: it biases the reported rate of CDI in surveillances data; it can lead to a delay in prescribing adequate therapy and consequently may cause a higher complication rate; it may contribute to an increase in the CDI transmission within hospitals due to the missed or delayed application of infection prevention and control measures, including patients’ isolation.

Essential actions aimed at reducing the rate of underdiagnosis are needed: 1. a further investigation of the current CDI epidemiology framework, especially to provide more detailed information on CA-CDI incidence and on its risk factors; 2. a better recognition of the differences in the rate of underdiagnosis reported between countries and between hospitals in the same countries [12]; 3. understanding the reasons for the lack of clinical suspicion; 4. tailoring initiatives for health-care workers to improve the accuracy and timeliness of the diagnosis.

The difficulties faced in reducing the spread of CD among hospitalized patients undeniably represent another relevant issue in CDI management. In the last years, a novel approach has been proposed, based on the hypothesis that asymptomatic CD carriers might represent a “submerged iceberg” that significantly contributes to the CD hospital spread.

It is well known that people could asymptomatically carry toxigenic CD strains. A meta-analysis found a pooled prevalence of toxigenic CD colonization at hospital admission of 8.1% [14]. CD-colonized patients may contaminate the surrounding environment and can be the source for patient-to-patient transmission, as is well documented [15].

A strategy of screening and isolation since admission has been largely applied for carriers of methicillin-resistant *Staphylococcus aureus*, carbapenem-resistant *Enterobacteriaceae* and vancomycin-resistant *Enterococci* [16,17,18]. Actually, few studies have already assessed the impact of colonized patients on the spread of CDI and on the effectiveness of placing CD carriers into contact precautions. Longtin and colleagues assessed the screening and isolation strategy for CD carriers and reported a significant decrease in the incidence of CDI [19]. Importantly, the change in incidence was not immediate, but there was a significant decrease in trend over time of 7% per four-week period. Interestingly, the intervention lead to a decrease in the proportion of CDI due to the NAP1 strain [19]. More recently, in another study patients were tested at admission if they reported hospitalizations in the previous two months, if they had a past CDI diagnosis, or if they were in a long-term care facility in the previous six months [20]. Eight per cent of tested patients were CD carriers. All colonized patients were placed on contact precautions and compliance with measures to reduce the incidence of HCA-CDI was assessed; as a consequence, a significant reduction in hospital-acquired incidence was observed (4.23 cases/10,000 patient days versus 5.96 cases/10,000 patient days in the period previous the introduction of the strategy, *p* = 0.02) [20].

This new approach for improving the prevention of hospital CD spread seems promising; however, the strategy needs to be evaluated in different healthcare settings. In particular, since the rate of CD-colonized patients at admission could vary widely between different wards and hospitals, it would be important to determine the cost-benefit and the cost effectiveness of this strategy in different epidemiological settings. In addition, the effectiveness of this strategy could be investigated in wards admitting patients with a higher risk of developing infections and having a worse outcome, such as oncology and haematology units, geriatric wards and intensive care units.

One of the most troublesome characteristics of CDI is represented by its capability to reoccur, in some cases several times in the same patient. CDI recurrences (rCDI) have a very strong impact on the patient quality of life, on the rate of re-hospitalization and on morbidity and mortality. According to the result of a meta-analysis, the risk of rCDI varies between 10.1% and 50.8% [21], with a 40% probability of a second recurrence episode [22].

The definition of recurrence is controversial; in fact, the eight-week cutoff of the rCDI definition [23] may be unable to distinguish reliably between recurrence and reinfection. Molecular typing studies of CD have provided insight into the rate of infections occurring after the first CDI episode that are caused by different or identical strains of CD and that can be more reliably defined as recurrences or reinfections. Recently, some authors reported that identical strains were identified during both episodes of CDI in 77% of patients with recurrence (36 of 47) and in 46% of patients with reinfection (27 of 59) [24]. Conversely, 23% of patients diagnosed with a second episode of CDI during the first eight weeks after initial infection were found to be infected with a different strain [24]. Whole genome sequencing has also been used to determine the relation of CD strains isolated from patients with recurrent CDI, reporting 16% of recurrent episode caused by a different CD strain [25]. On the other hand, it must be considered that mixed CDI has been described [26,27], with more than one CD genotype detected in each CDI episode.

The differentiation between recurrence and reinfection has important implications both in the study of pathogenesis of rCDI and in the assessment of the effectiveness of a treatment to prevent rCDI. Therefore, assessing the efficacy of an anti-CDI treatment in preventing recurrences on the basis of a temporal period could lead to an over- or underestimate of the effect of the treatment itself, and comparisons should take into account the CD genotypes.

It is still not clear which is the pathway leading to recurrence, even if a role of intestinal microbiota disruption, i.e., dysbiosis, is undeniable. Three meta-analysis investigated the most common risk factors associated with rCDI [21,28,29]. Regrettably, several shortcomings affected the studies included in the meta-analyses: the study design; different diagnostic tests used to detect CDI; heterogeneity in definition of rCDI and follow-up period. Obviously, these shortcomings importantly affect our knowledge of rCDI.

Additionally, data on the real incidence of rCDI and its outcome are still limited. Very large prospective cohort studies including patients with a first CDI episode and following them for more than eight weeks after CDI diagnosis are therefore urgently needed. These studies should include patients with different risk factors for rCDI, i.e., antibiotic exposure, renal impairment, older age and repeated exposure to healthcare settings in order to assess the added impact of other risk factors in each sub-cohort of patients. In addition, these studies could allow for reaching a better estimation of the incidence of rCDI in different sub-cohorts in order to tailor prevention interventions.

Finally, the management of CDI in terms of prevention and treatment is well established, but some needs are still unmet. Indeed, new antibiotics to resolve acute CDI should be able also to reduce recurrences, and the preventive measures either for acute and recurrent CDI should be based on a multifaceted approach that includes: a better use of current anti CD antibiotics (i.e., taper and pulsing dosing); the introduction of very narrow spectrum anti-CD molecules; a more favourable timing for the use of passive antibodies; minimizing intestinal dysbiosis during an antibiotic course; restoring gut microbioma with effective and easily feasible approaches using biotherapeutics, including manufactured faecal microbiota transplant derivatives and non-toxigenic *C. difficile*, up to the introduction of vaccines targeted at CD toxins [30].

An essential step toward zeroing rCDI should be represented by the investigation of new treatment options for rCDI in special population groups such as patients needing antibiotic treatment for concomitant bacterial infections, patients affected by inflammatory bowel disease or cancer, and elderly people. Importantly, it should be ascertained the usefulness of prescribing these therapies to patients with a first CDI episode.

In conclusion, the experience of COVID-19 has taught everyone that nothing should be taken for granted. Stewardship, infection prevention and control do not have to be just catchphrases. More insight should be given to epidemiological issues; recurrences should be faced with multifaceted competencies; and new treatments should better respect the ecology of gut microbioma.

For CDI, a continuous commitment is needed to respond to unmet needs and to settle unsolved questions.
